# Overcoming restriction as a barrier to DNA transformation in *Caldicellulosiruptor* species results in efficient marker replacement

**DOI:** 10.1186/1754-6834-6-82

**Published:** 2013-05-29

**Authors:** Daehwan Chung, Joel Farkas, Janet Westpheling

**Affiliations:** 1 Department of Genetics, University of Georgia, Athens, GA 30602, USA; 2 The BioEnergy Science Center, Oak Ridge National Laboratory, Oak Ridge, TN 37831, USA

**Keywords:** *Caldicellulosiruptor* species, Biomass conversion, Restriction-modification enzymes, CbeI, M.CbeI, Targeted deletion

## Abstract

**Background:**

Thermophilic microorganisms have special advantages for the conversion of plant biomass to fuels and chemicals. Members of the genus *Caldicellulosiruptor* are the most thermophilic cellulolytic bacteria known. They have the ability to grow on a variety of non-pretreated biomass substrates at or near ~80°C and hold promise for converting biomass to bioproducts in a single step. As for all such relatively uncharacterized organisms with desirable traits, the ability to genetically manipulate them is a prerequisite for making them useful. Metabolic engineering of pathways for product synthesis is relatively simple compared to engineering the ability to utilize non-pretreated biomass.

**Results:**

Here we report the construction of a deletion of *cbeI* (Cbes2438), which encodes a restriction endonuclease that is as a major barrier to DNA transformation of *C. bescii*. This is the first example of a targeted chromosomal deletion generated by homologous recombination in this genus and the resulting mutant, JWCB018 (Δ*pyrFA* Δ*cbeI*), is readily transformed by DNA isolated from *E. coli* without *in vitro* methylation. PCR amplification and sequencing suggested that this deletion left the adjacent methyltransferase (Cbes2437) intact. This was confirmed by the fact that DNA isolated from JWCB018 was protected from digestion by CbeI and HaeIII. Plasmid DNA isolated from *C. hydrothermalis* transformants were readily transformed into *C. bescii*. Digestion analysis of chromosomal DNA isolated from seven *Caldicellulosiruptor* species by using nine different restriction endonucleases was also performed to identify the functional restriction-modification activities in this genus.

**Conclusion:**

Deletion of the *cbeI* gene removes a substantial barrier to routine DNA transformation and chromosomal modification of *C. bescii*. This will facilitate the functional analyses of genes as well as metabolic engineering for the production of biofuels and bioproducts from biomass. An analysis of restriction-modification activities in members of this genus suggests a way forward to eliminating restriction as a barrier to DNA transformation and efficient genetic manipulation of this important group of hyperthermophiles.

## Background

Biomass recalcitrance represents the greatest obstacle to the efficient conversion of lignocellulosic biomass to commodity chemicals and biofuels [1-3]. For this reason, thermophilic cellulolytic bacteria that are capable of degrading and utilizing plant biomass are of special interest. Members of the *Caldicellulosiruptor* genus are able to utilize several plant-derived substrates efficiently, including unpretreated switchgrass, and are the most thermophilic of the cellulolytic bacteria (optimum growth temperature near 80°C) [4-6]. These species accomplish plant biomass degradation by producing an arsenal of extracellular carbohydrate degrading enzymes [4,7,8] that include cellulases with multiple catalytic enzyme modules in a single multi-domain enzyme. This is distinct from, but somewhat similar to, membrane-bound cellulosomes exemplified by *Clostridium thermocellum* and other anaerobes [5,8-11]. Recent growth experiments on crystalline cellulose (Avicel) revealed a significant disparity in plant cell wall deconstruction capability among eight sequenced *Caldicellulosiruptor* species [4]. These distinctive features provide a unique opportunity for the identification of enzymes that facilitate plant biomass decomposition, as well as the basis for a better understanding of the mechanisms of crystalline cellulose degradation.

The development of *Caldicellulosiruptor* species for “consolidated bioprocessing” (CBP) [12] has been limited by the lack of genetic tools required to create stable strains with high yields of desired biofuels and/or bioproducts. Recently, we reported methods for efficient DNA transformation of *C. bescii* and *C. hydrothermalis*, including transformation of a shuttle vector [13] and the ability to direct marker replacement between non-replicating plasmids and chromosomal genes [14]. Restriction by CbeI was shown to be an absolute barrier to DNA transformation [15], but could be overcome by *in vitro* methylation of DNA by a cognate methyltransferase, M.CbeI [14].

Restriction-modification (R-M) systems were initially identified in *Escherichia coli* nearly 6 decades ago [16,17] and are now known to be wide spread in bacteria and archaea. Almost 90% of bacterial genomes contain R-M systems and 43% contain four or more according to “The Restriction Enzyme Database” (REBASE) [18]. R-M systems comprise pairs of distinctive enzymatic activities, a restriction endonuclease and a DNA methyltransferase. R-M systems are classified as type I, type II, type IIS, type III and type IV according to enzyme composition, cofactor requirements, recognition sequence symmetry, location of DNA cleavage relative to the recognition site, and mode of action [19]. They provide the best-characterized defense mechanism in prokaryotes - a “self-nonself discrimination”, against invasion of foreign DNA that includes phages or conjugative plasmids [20,21]. The methyltransferase subunits of R-M systems methylate specific sites in the host DNA (“self”) thus preventing cleavage by the cognate restriction endonuclease. Nonmethylated foreign DNA (“nonself”) is cleaved by the restriction endonuclease [22]. R-M systems also constitute a formidable barrier to efficient DNA transformation for genetic manipulation, especially DNA from other genera, most notably, *E. coli*. Eliminating restriction endonuclease activities in a number of host organisms, including *Bacillus subtilis*[23], *Thermosynechococcus elongates*[24], *Borrelia burgdorferi*[25], and *Clostridium acetobutylicum*[26], improved transformation efficiency and simplified the transformation protocols by removing the time-consuming laborious DNA modification steps.

Here we show that deletion of the gene encoding the CbeI (Cbes2438) restriction endonuclease resulted in a strain that is easily transformable with unmethylated DNA from *E. coli*, eliminating the need for *in vitro* methylation by M.CbeI (Cbes2437). CbeI is a type II restriction endonuclease that recognizes the sequence 5′-GGCC-3′ [15]. We further extend the current study to the analysis of chromosomal DNA modification in other species of *Caldicellulosiruptor*, showing that the pattern of DNA modification is quite diverse across the genus. Both GenBank [27] and REBASE [18] predicted that all 8 sequenced *Caldicellulosiruptor* species [28-33] contain a large number of R-M systems. While the isolation or construction of restriction-deficient strains for all members of this genus is impractical at this time, plasmid DNA from *C. hydrothermalis*, which appears to have a similar R-M system to *C. bescii*, is transformable to *C. bescii* without additional modification. We present a strategy for transformation and genetic manipulation of the other species within the *Caldicellulosiruptor* genus.

## Results and discussion

### Restriction digestion analysis of chromosomal DNA from *Caldicellulosiruptor* species

We previously reported that the restriction endonuclease, CbeI, presents an absolute barrier to transformation of *C. bescii*[15] with DNA isolated from *E. coli*, and this was successfully overcome by *in vitro* methylation of transforming DNA with M.CbeI, the cognate methyltransferase [14]. The observation that restriction was an absolute barrier to DNA transformation of *C. bescii* prompted us to investigate the prevalence of functional R-M systems in other *Caldicellulosiruptor* species. The finding that the M.CbeI methylated DNA successfully transformed *C. hydrothermalis*[13] suggested that *C. hydrothermalis* and *C. bescii* might share similar R-M activities. A large number of putative R-M systems with significant variation were detected in *Caldicellulosiruptor* species based on REBASE [18] and GenBank [27] analysis. To address the issue of which, if any, of these R-M systems are functional, chromosomal DNA was isolated from 7 *Caldicellulosiruptor* species and digested with each of 9 different restriction endonucleases, all of which have commercially available cognate methyltransferases (Table 1 and Additional file 1: Figure S1). We found that all species tested contain at least 3 types of functional R-M systems (Table 1). DNA isolated from each of the 7 species was resistant to digestion by BamHI and BspEI, indicating the presence of a cognate methyltransferase for these restriction endonucleases is common in this genus. Resistance to digestion by HaeIII was observed for *C. bescii, C. hydrothermalis, C. kristjansonii,* and *C. saccharolyticus*. Resistance to digestion by MboI was observed for *C. kristjansonii, C. saccharolyticus, C. obsidiansis, C. lactoaceticus,* and *C. kronotskyensis*. HaeIII (5′-GGCC-3′) and MboI (5′-GATC-3′) would be expected to act as a formidable barrier for DNA transformation from *E. coli* for these species, since both enzymes are four base cutters and are known to be absolute barriers to DNA transformation in other microorganisms [15,34,35]. *C. kronotskyensis* appears to be the most different from the other species in terms of R-M systems, as it has apparent methyltransferase activity specific to HpaII and MspI recognition sites. All 7 tested species were sensitive to digestion by AluI, EcoRI, and HhaI (Table 1). In this limited test, we observed no differences between *C. bescii* and *C. hydrothermalis* and, in fact, plasmid DNA from *E. coli* methylated *in vitro* with M.CbeI readily transformed both *C. hydrothermalis* and *C. bescii*[13,14]. Both *C. kristjansonii* and *C. saccharolyticus* were very similar to *C. bescii* and *C. hydrothermalis,* differing only by one cognate methyltransferase activity (Table 1). This preliminary analysis with the nine enzymes tested has already provided access to genetic manipulation of *C. hydrothermalis* and while it is possible, if not likely, that other functional R-M systems exist, these data provide insight into extending genetic technologies to other members of this genus.

**Table 1:** **Susceptibility of *Caldicellulosiruptor *species chromosomal DNA to restriction endonuclease digestion**

**Source**	**Restriction endonucleases used for digestion**								
	**AluI**	**BamHI**	**BspEI**	**EcoRI**	**HaeIII**	**HhaI**	**HpaII**	**MboI**	**MspI**
*C. bescii* DSM6725	+	-	-	+	-	+	+	+	+
*C. hydrothermalis* DSM 18901	+	-	-	+	-	+	+	+	+
*C. kristjansonii* DSM12137	+	-	-	+	-	+	+	-	+
*C. saccharolyticus* DSM8903	+	-	-	+	-	+	+	-	+
*C. obsidiansis* ATCC BAA-2073	+	-	-	+	+	+	+	-	+
*C. lactoaceticus* DSM9545	+	-	-	+	+	+	+	-	+
*C. kronotskyensis* DSM12137	+	-	-	+	+	+	-	-	-

“+” succeptible to digestion; “-” resistance to digestion.

### Construction of a *cbeI* deletion in *C. bescii*

**Figure 1:**
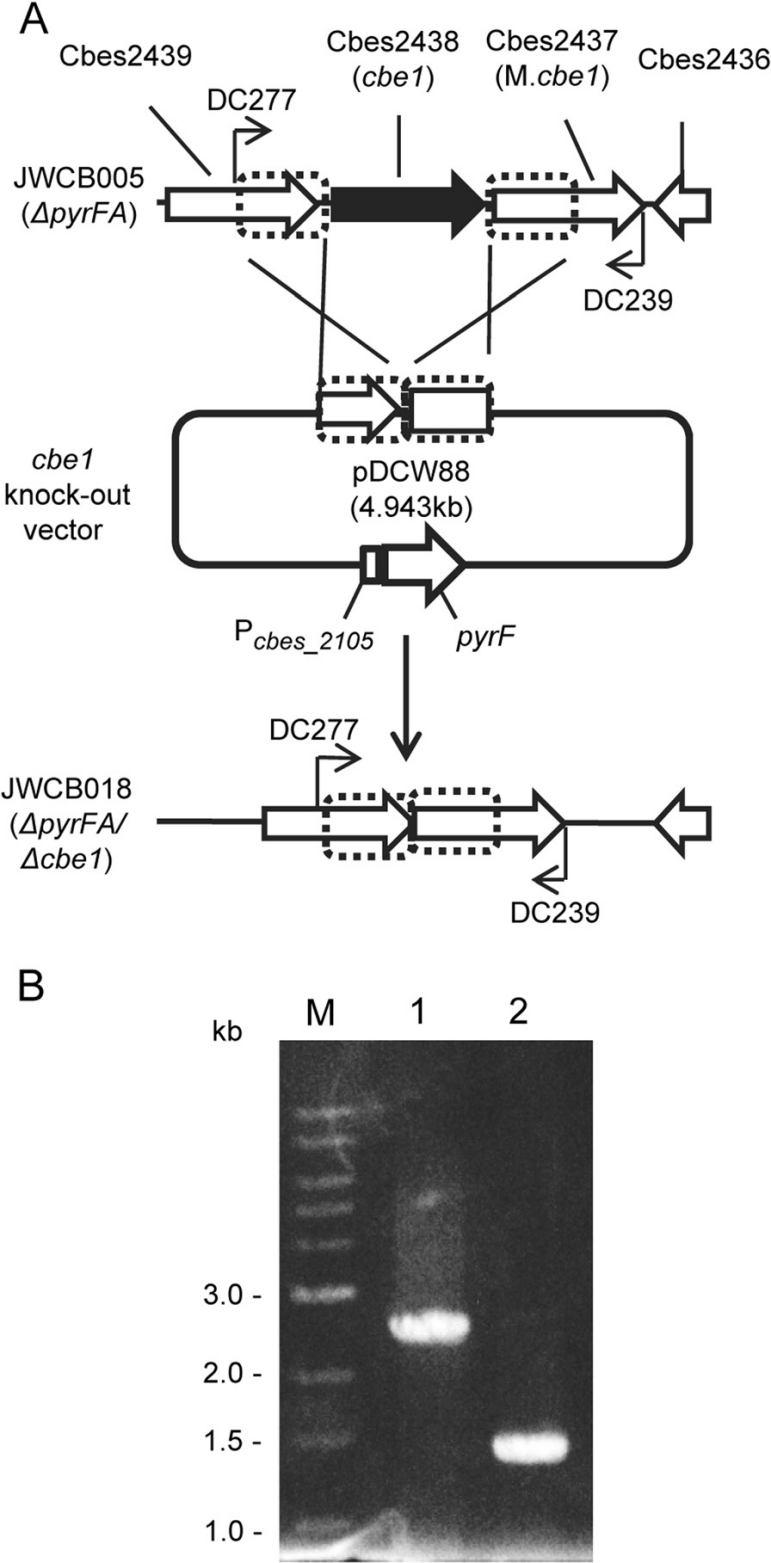
**Strategy for construction and PCR analysis of the *cbeI *(Cbes2438) deletion in JWCB005.** (**A**) A diagram of the *cbeI* genome region is shown with the *cbeI* knock-out plasmid having ~ 0.5 kb regions from each up- and downstream of *cbeI* for homologous recombination and also containing the *pyrF* cassette [13] for selection of transformants. Homologous recombination can occur at the upstream or downstream *cbeI* flanking regions, integrating the plasmid into the genome and generating a strain that is a uracil prototroph. Counter-selection with 5-fluoroorotic acid (5-FOA) selects for loss of the plasmid and deletion of the *cbeI* gene. Bent arrows depict primers used for verification of the *cbeI* deletion. (**B**) Gel depicting PCR products amplified from the *cbeI* genome region in JWCB018 (Δ*pyrFA*/Δ*cbeI*) compared to the parental strain JWCB005 (Δ*pyrFA*), amplified by primers (DC277 and DC239). Lane 1: JWCB005; lane 2: JWCB018; M: 1 kb DNA ladder (NEB).

Transformation of DNA from *E. coli* to *C. bescii* required *in vitro* methylation of the DNA with M.CbeI, which was expressed and purified from *E. coli*, requiring a considerable amount of time and effort [14]. More importantly, the degree of methylation *in vitro* had a profound effect on the transformation efficiency introducing an element of variation in the method. CbeI was, therefore, an obvious first target for a chromosomal deletion in *C. bescii*. To test whether a deletion of *cbeI* would alleviate restriction of DNA from *E. coli* in *C. bescii* and allow transformation of unmethylated DNA, we constructed a chromosomal deletion of *cbeI* (Cbes2438) in JWCB005 (Figure 1A, Table 2) [13], using a targeted marker replacement strategy previously described [14]. Strain JWCB005 (Δ*pyrFA, ura*^*-*^*/5-FOA*^*R*^) previously shown to be suitable for nutritional selection and counter-selection for 5-fluoroorotic acid (5-FOA) [13] was used as a host strain. The *cbeI* knock-out vector, pDCW88, contains a 927 bp DNA fragment that includes both the 5′ (440 bp) and 3′ (487 bp) flanking regions of *cbeI*, and the wild type *pyrF* cassette [13] for uracil prototrophic selection of transformants (Figure 1A, Additional file 1: Figure S2). This non-replicating vector in *C. bescii* was transformed into JWCB005 (*ΔpyrFA*) with selection for uracil prototrophy followed by counter-selection for 5-fluoroorotic acid (5-FOA) resistance. Initial screening of 18 isolates by PCR revealed merodiploids with a mixture of wild type and *cbeI* deletion genomes. Three of these were further purified on solid medium without 5-FOA and analyzed by PCR amplification of the *cbeI* locus in the chromosome with primers DC277 and DC239 (Figure 1). PCR amplification of this locus from the parent strain JWCB005 (*ΔpyrFA*) produced the expected wild type ~2.4 kb band, while amplification from JWCB018 produced a ~1.4 kb band indicating a deletion within this region (Figure 1B). The site of the deletion was confirmed by DNA sequence analysis of the PCR product. The resulting strain, JWCB018 (*ΔpyrFA ΔcbeI*) (Table 2) was used for further analysis.

**Table 2:** Strains and plasmids used in this study

**Strains or plasmid**	**Strain and genotype/phenotype**	**Source**
*Caldicellulosiruptor*		
*C. bescii* DSM6725	Wild type / (ura^+^/5-FOA^S^)	DSMZ^1^
*C.hydrothermalis* DSM18901	Wild type / (ura^+^/5-FOA^S^)	DSMZ^1^
*C. kristjansonii* DSM12137	Wild type / (ura^+^/5-FOA^S^)	DSMZ^1^
*C. saccharolyticus* DSM8903	Wild type / (ura^+^/5-FOA^S^)	DSMZ^1^
*C. obsidiansis* ATCC BAA-2073	Wild type / (ura^+^/5-FOA^S^)	DSMZ^1^
*C. lactoaceticus* DSM9545	Wild type / (ura^+^/5-FOA^S^)	DSMZ^1^
*C. kronotskyensis* DSM12137	Wild type / (ura^+^/5-FOA^S^)	DSMZ^1^
JWCB005	*C. bescii *Δ*pyrFA* / (ura^-^/5-FOA^R^)	[13]
JWCB018	*C. bescii *Δ*pyrFA*Δ*cbeI* / (ura^-^/5-FOA^R^)	This study
JWCH003	*C. hydrothermalis* IS*cahyI* insertion mutation in *pryF* gene / (ura^-^/5-FOA^R^)	[13,36]
JWCH005	JWCH003 transformed with M.CbeI methylated pDCW89 / (ura^+^/5-FOA^S^)	[13]
*Escherichia coli*		
JW291	DH5α containing pDCW88 (Apramycin^R^)	This study
JW292	DH5α containing pDCW89 (Apramycin^R^)	[13]
Plasmids		
pDCW88	*cbeI* kcock-out vector (Apramycin^R^)	This study
pDCW89	*E. coli/Caldicellulosiruptor* species shuttle vector (Apramycin^R^)	[13]

^1^German collection of microorganisms and cell cultures.

The *cbeI* gene is located in the chromosome adjacent to the gene encoding M.CbeI, its cognate methyltransferase [14,15]. The two genes are separated by only 45 bases, and are likely to be transcriptionally coupled. The deletion of *cbeI* spanned the entire *cbeI* coding region, but left the potential regulatory region upstream intact, and deleted only 23 bases of the downstream flanking region leaving the entire M.CbeI coding region intact. Chromosomal DNA isolated from JWCB018 was completely protected from cleavage by HaeIII and CbeI *in vitro* (data not shown), suggesting that M.CbeI is still functional in JWCB018. Growth of this mutant was comparable to growth of the parent JWCB005 and the wild type strain.

### The JWCB018 is efficiently transformed with unmethylated DNA

**Figure 2:**
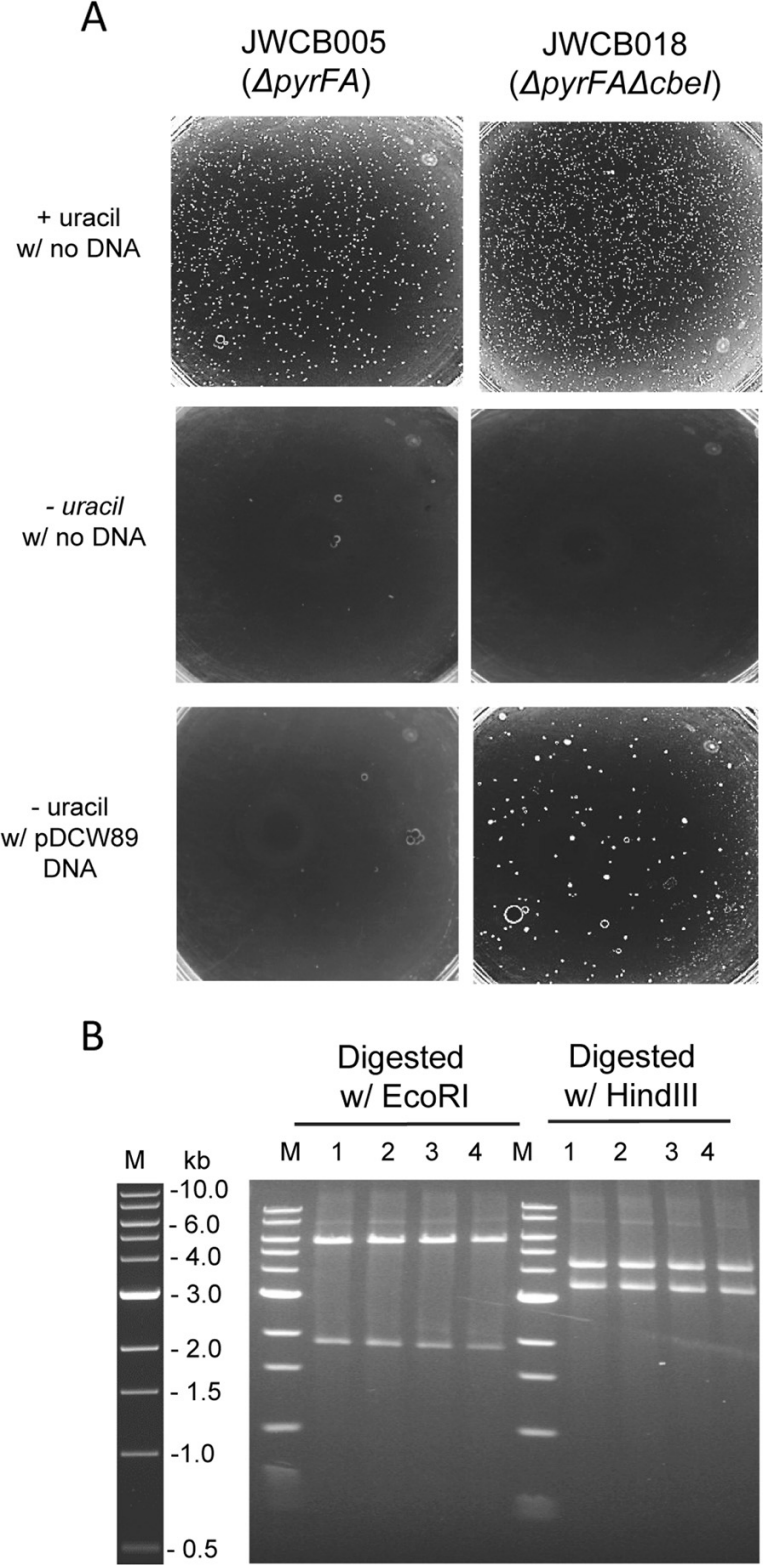
**Electrotransformation of unmethylated pDCW89 into JWCB018.** (**A**) JWCB005 (Δ*pyrFA*) and JWCB018 (Δ*pyrFA*Δ*cbeI*) were transformed with un-methylated pDCW89 DNA and plated onto defined medium either with or without uracil. Controls with no DNA transformation are also presented. (**B**) Restriction analysis of pDCW89 plasmid DNA before and after transformation of *C. bescii* and back-transformation to *E. coli*. Lane 1, pDCW89 plasmid DNA isolated from *E. coli DH5α*, digested with either EcoRI (5.8 kb and 1.9 kb cleavage products), or with HindIII (4.4 kb and 3.3 kb cleavage products). Lanes 2, 3 and 4 plasmid DNA isolated from three biologically independent *E. coli DH5α* back-transformants using total DNA isolated from *C. bescii* transformants, digested with either EcoRI or HindIII. M: 1 KB DNA ladder (NEB).

To assess the effect of the *cbeI* deletion on transformation of *C. bescii* with unmethylated DNA from *E. coli*, JWCB005 (Δ*pyrFA*) and JWCB018 (Δ*pyrFA* Δ*cbeI*) were transformed with unmethylated pDCW89 DNA, using a recently developed replicating shuttle vector [13] containing a wild type copy of the *pyrF* allele for uracil prototrophic selection (Figure 2). No transformants of the parent strain, JWCB005, were detected using unmethylated plasmid DNA isolated from *E. coli* (<10^-8^ transformants per μg plasmid DNA). The *ΔcbeI* strain, however, was readily transformed with unmethylated pDCW89 DNA isolated from *E. coli* (~1.0 × 10^3^ transformants per μg of plasmid DNA, Figure 2A). Methylated plasmid DNA transformed into the parent strain (JWCB005) at a frequency (~0.5 × 10^3^ transformants per mg plasmid DNA) and the difference is likely due to incomplete methylation of the plasmid DNA *in vitro*. Transformation of *C. bescii* was initially confirmed by PCR amplification of the pSC101 *E. coli* replication origin fragment present only in the plasmid (data not shown). Isolation of large quantities of pDCW89 from *C. bescii* for direct analysis proved to be difficult, likely due to low copy number as a result of competition with the endogenous plasmid, pBAS2 [13,37]. Total DNA isolated from JWCB018 transformants was used to “back-transform” *E. coli* and plasmid DNA isolated from these back-transformants was analyzed by restriction digestion (Figure 2B). pDCW89 DNA isolated from the “back transformants” was indistinguishable from the pDCW89 used to transform *C. bescii* and showed no obvious signs of rearrangement or deletion through transformation into JWCB018, replication in *C. bescii* or back-transformation to *E. coli* (Figure 2B).

### Plasmid DNA isolated from *C. hydrothermalis* readily transforms strain JWCB005 (Δ*pyrFA)* without *in vitro* methylation

**Figure 3:**
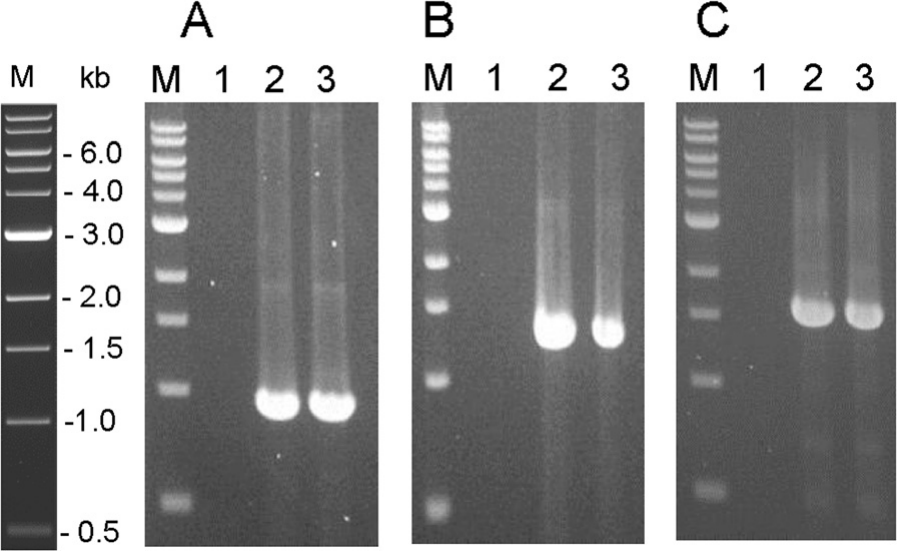
**PCR analysis for the presence of pDCW89 after transformation into JWCB005.** pDCW89 DNA isolated from *C. hydrothermalis* transformants was used to transform *C. bescii* (Δ*pyrFA*) and the presence of the plasmid was confirmed by PCR amplification of sequences contained only on the plasmid (**A**) the 0.9 kb PCR product of *aac* (Apramycin Resistance gene cassette), (**B**) the 1.3 kb PCR product of *pyrF* cassette and (**C**) the 1.6 kb PCR product of *pSC101 ori* region. Lane 1: total DNA isolated from JWCB005; lane 2: total DNA isolated from *C. bescii* transformant; lane 3: pDCW 89 isolated from *E. coli*. M: 1 kb DNA ladder (NEB).

Given the fact that *C. hydrothermalis* and *C. bescii* showed the same observed functional R-M activities (Table 1), we anticipated that DNA isolated from *C. hydrothermalis* would be methylated by its homologue of M.CbeI (Calhy0409) and that plasmid DNA isolated from *C. hydrothermalis* might transform *C. bescii* without *in vitro* methylation. Plasmid DNA was readily isolated from *C. hydrothermalis* transformants (perhaps indicating a high copy number in *C. hydrothermalis* as it is derived from a high copy number *C. bescii* native plasmid [13,37]) and used to transform *C. bescii*. Transformants were obtained at frequencies comparable to M.CbeI methylated plasmid (~0.5 × 10^3^ per mg of plasmid DNA). The presence of pDCW89 in transformants was confirmed using PCR amplification of the *aac* (apramycin resistance gene), pSC101 *ori* region, and *pyrF* cassette, contained only on the plasmid. The size of the PCR products obtained in this analysis were as expected and were generated from total DNA isolated from the JWCB005 transformants and plasmid DNA isolated from *E.coli*, but not from JWCB005 (Figure 3). Total DNA, isolated from JWCB005 transformants, was back-transformed to *E. coli* for further analysis. Restriction analysis of plasmid DNA isolated from back-transformants showed that pDCW89 was structurally stable through transformation and replication in *C. bescii* (data not shown).

## Conclusions

Here we report the construction of a deletion of *cbeI* (Cbes2438), which encodes a potent restriction endonuclease whose activity is apparently an absolute barrier to transformation of DNA from *E. coli* to *C. bescii*. We recently showed that *in vitro* methylation of DNA from *E. coli* with M.cbeI, a cognate methyltransferase from *C. bescii*, allowed transformation. Deletion of *cbeI* is the first targeted deletion in this genus and the resulting mutant, JWCB018 (*ΔpyrFA ΔcbeI*), is readily transformed by DNA isolated from *E. coli* without *in vitro* methylation. PCR amplification and sequencing suggested that this deletion left the adjacent methyltransferase (M.CbeI) intact and its function was confirmed by the fact that chromosomal DNA isolated from JWCB018 was protected from digestion by CbeI and HaeIII *in vitro*.

The construction of this mutant strain removes a substantial barrier to routine transformation and chromosomal modification and is substantially more efficient than other possible methods including modification of the vector prior to transformation, using engineered vectors containing no or fewer restriction sites recognized by restriction endonuclease in host [38,39], conditional inactivation of the R-M systems [40,41] or using group II intron insertion technology to disrupt a restriction system in *Clostridum acetobutylicum*[26]. The ability demonstrated here to make targeted gene deletions is a powerful and direct tool for the investigation of *in vivo* gene function and the deletion of this endonuclease resulted in a strain that will provide the basis for further genetic manipulation. The combined efficiencies of transformation and homologous recombination (with as few as 450 bp of homology) in *C. bescii* allow us to use non-replicating plasmids for genetic manipulation. This is fortuitous and a significant benefit for the development of *Caldicellulosiruptor* species as CBP organisms. The proven CBP microbe, *Clostridium thermocellum*, for example, is genetically tractable but the efficiency of transformation and/or recombination does not permit the use of non-replicating plasmids for marker replacement, significantly extending the time required for mutant construction [42].

Plasmid DNA isolated from *C. hydrothermalis* was able to transform *C. bescii* JWCB005 (*ΔpyrFA*) and *C. bescii* JWCB018 (*ΔpyrFAΔcbeI)*, however total DNA isolated from the *C. bescii* transformant did not transform *C. hydrothermalis*. This apparent contradiction could be due to the low concentration of pDCW89 in total DNA isolated from the *C. bescii* transformant [13]. Alternatively, this may be due to yet another unidentified R-M system difference between these species. REBASE [18] predicts that the genome of *C. bescii* encodes as many as six methyltransferases in addition to M.CbeI and that *C. hydrothermalis* contains as many as six restriction endonucleases, one of which (Calhy0018) is a type IV (methyl-directed) restriction endonuclease not present in *C. bescii*. If this type IV restriction endonuclease is active and recognizes methylated DNA formed by a *C. bescii* methyltransferase not present in *C. hydrothermalis*, the DNA isolated directly from *C. bescii* would be cleaved. Somewhat surprisingly, the disruption of only one functional restriction enzyme was enough to overcome restriction as a barrier to transformation of *C. bescii* by *E. coli* DNA. This may be due to the fact that BamHI and BspEI are rare cutters in *C. bescii* genomic DNA and neither of these sites is present in pDCW88 used to make the deletion of *cbeI*. Only one BspEI site is present in the pDCW89 shuttle vector and that may be protected due to an overlapping *dam* site created by *in vivo* methylation in *E. coli* DH5α. It is also possible that the cognate restriction endonuclease of the BspEI methyltransferase is inactive or absent altogether.

Perhaps the most powerful conclusion to be drawn from these data is the direction forward in overcoming R-M systems as a barrier to transformation of other *Caldicellulosiruptor* species. As shown in *C. bescii*, a 4 base cutter such as CbeI (5′-GGCC-3′) is a formidable obstacle to transformation. In contrast, 6 base cutters can be easily avoided during plasmid construction (as with BamHI and BspEI here), as long as the functional R-M system is known. Based on our findings, all *Caldicellulosiruptor* species show at least three DNA methyltransferase activities (Table 1), though it is not known that all modification activities are paired with a cognate restriction activity. *In vitro* methylation of plasmid DNA with cell-free extracts may be useful going forward to identify restriction activities that present obstacles to transformation. In any case, these observations emphasize that successful transformation is largely empirical. It was perhaps fortunate that *C. bescii* was the first chosen for genetic manipulation, because only one methyltransferase activity was required for efficient transformation of DNA from *E. coli*.

## Methods

### Strains, media and growth conditions

*Caldicellulosiruptor* and *E. coli* strains used in this study are listed in Table 2. All *Caldicellulosiruptor* species were grown anaerobically in liquid or on solid surface in either modified DSMZ 516 medium [14] or in low osmolarity defined (LOD) medium [43] with maltose as the carbon source. *C. bescii, C. kristjansonii,* and *C. obsidiansis* were incubated at 75°C. *C. hydrothermalis, C. kronotskyensis, C. lactoaceticus,* and *C. saccharolyticus* were incubated at 68°C. For growth of auxotrophic mutants, the defined medium contained 40 μM uracil. *E. coli* strain DH5α was used for plasmid DNA constructions and preparations. Standard techniques for *E. coli* were performed as described [44]. *E. coli* cells were grown in LB broth supplemented with apramycin (50 μg/mL) and plasmid DNA was isolated using a Qiagen Mini-prep Kit. Chromosomal DNA from *Caldicellulosiruptor* strains was extracted using the Quick-gDNA™ MiniPrep (Zymo) or using the DNeasy Blood & Tissue Kit (Qiagen) according to the manufacturer’s instructions. Plasmid DNA isolation from *Caldicellulosiruptor* species was performed as described [15].

### Construction of pDCW88

A 927 bp DNA fragment containing the 5′ flanking region (440 bp) and the 3′ flanking region (487 bp) of *cbeI* (Cbes2438) was generated by overlap extension polymerase chain reaction (OE-PCR) using primers DC265 (with KpnI site), DC266, DC267, and DC268 (with ApaLI site). All PCR reactions were performed using *pfu* turbo (Agilent Technologies), and *C. bescii* genomic DNA as a template. The DNA fragments containing the apramycin resistance gene cassette, *pyrF* cassette, and the *E. coli pSC101* replication origin, were amplified from pDCW 89 [13] using primers DC081 (with KpnI site) and DC262 (with ApaLI site). These two linear DNA fragments were digested with KpnI and ApaLI, and ligated to generate pDCW88 using Fast-link DNA Ligase kit (Epicentre Biotechnologies) according to the manufacturer’s instructions. DNA sequences of the primers are shown in Additional file 1: Table S1. A diagram of pDCW88 is shown in Additional file 1: Figure S2. *E. coli* strain DH5α cells were transformed by electroporation in a 2-mm-gap cuvette at 2.5 V and transformants were selected for apramycin resistance. The sequence of pDCW88 was confirmed by Automatic sequencing (Macrogen USA, Maryland). All plasmids are available on request.

### Screening, purification, and sequence verification of deletion mutants

To construct strain JWCB018, one microgram of M.CbeI methylated pDCW88 DNA was used to electrotransform JWCB005 (*ΔpyrFA*) as described [14]. Cells were then plated onto solid defined medium (without uracil and casein) and uracil prototrophic transformant colonies were inoculated into liquid medium for genomic DNA extraction and subsequent PCR screening of the targeted region. Confirmed transformants were inoculated into nonselective liquid defined medium, with 40 μM uracil, and incubated overnight at 75°C to allow loop-out of the plasmid DNA. The cultures were plated onto 5-FOA (8 mM) containing solid medium. After initial screening, transformants containing the expected deletion were further purified by three additional passages under selection on solid medium and screened a second time by PCR to check for segregation of the deleted allele. The deletions were then verified by PCR amplification and sequence analysis. A PCR product was generated from genomic DNA by using primers (DC277 and DC239) outside the homologous regions used to construct the deletion, and internal primers were used to sequence the PCR product. For PCR, the extension time was sufficient to allow amplification of the wild-type allele, if it were still present. Another set of primers, one located inside of the Cbes2438 open reading frame, and the other located outside of the flanking region were used for further verification. Growth of this strain, JWCB018, supplemented with uracil (40 μM) was comparable to wild type reaching a cell density of ~2 × 10^8^ in 20 hours. Cells were counted in a Petroff Hausser counting chamber using a phase-contrast microscope with 40X magnification.

### Transformation of *C. bescii* and selection of transformants

Electrotransformations of JWCB005 and JWCB018 with unmethylated pDCW89 from *E. coli* or isolated plasmid DNA from *C. hydrothermalis* transformants were performed as described [14]. For selection of transformants, after electro-pulse the recovery cultures with pDCW89 DNA (0.5 – 1.0 μg) were plated onto the defined medium without casein and uracil. Uracil prototrophic transformants were inoculated into liquid medium for DNA isolation. The presence of plasmid sequences in *C. bescii* transformants was confirmed by PCR amplification of the *aac* (apramycin resistance gene cassette) gene, the pSC101 *ori* region, and the *pyrF* cassette, present only on pDCW89. The transformation frequencies reported herein take into account the number of cells plated as determined by culture cell counts (this does not take into account the plating efficiency), and, where indicated, the total amount of DNA added (i.e., the number of transformants per microgram of DNA). *E. coli* strain DH5α cells were used for back-transformation.

### Restriction endonuclease digestion of *Caldicellulosiruptor* species chromosomal DNA

Chromosomal DNA isolated from seven *Caldicellulosiruptor* species was subjected to digestion with the REs AluI, BamHI, BspEI, EcoRI, HaeIII, HhaI, HpaII, MboI, and MspI. All enzymes were from New England Biolabs. For each reaction, 1 microgram of DNA was incubated with the enzyme and appropriate buffer for 1 hour according to the manufacturer’s instructions. After incubation, digestion patterns were compared by electrophoresis on a 1.0% agarose gel.

## Abbreviations

CBP: Consolidated bioprocessing; C. bescii: *Caldicellulosiruptor bescii*; C. hydrothermalis: *Caldicellulosiruptor hydrothermalis*; C. kristjansonii: *Caldicellulosiruptor kristjansonii*; C. saccharolyticus: *Caldicellulosiruptor saccharolyticus*; C. obsidiansis: *Caldicellulosiruptor obsidiansis*; C. lactoaceticus: *Caldicellulosiruptor lactoaceticus*; C. kronotskyensis: *Caldicellulosiruptor kronotskyensis*; R-M: Restriction-modification; REBASE: Restriction Enzyme Database; 5-FOA: 5-fluoroorotic acid; LOD: Low osmolarity defined; Aac: Apramycin resistance gene cassette; LB: Luria broth.

## Competing interests

The authors declare that they have no competing interests.

## Authors’ contributions

DC designed and carried out the overall experiments, analyzed results and wrote the manuscript. JF participated in its design and coordination, carried out the restriction analysis, and drafted the manuscript. JW helped conceive of the study, participated in its design and coordination, and helped to draft and reviewed the manuscript. All authors read and approved the final manuscript.

## Supplementary Material

Additional file 1: Figure S1 Restriction endonuclease digests of chromosomal DNA isolated from *Caldicellulosiruptor* species. The nine restriction enzymes employed in this analysis are indicated on the top of the gel. (**A**) *C. bescii* chromosomal DNA. (**B**) *C. saccharolyticus* chromosomal DNA. M: 1 kb DNA ladder (NEB). **Figure S2.** Diagram of the *cbeI* (Cbes2438) knock-out vector. The gray colored boxes indicate sequences originating from *C. bescii*. Restriction sites and primers are indicated. aac, apramycin resistant gene cassette; pSC101, low copy replication origin in *E. coli*; *repA* and *par*, plasmid-encoded genes required for pSC101 replication and partition. **Table S1.** Primers used in this study. Click here for file
