# Supplementary material for: Overcoming restriction as a barrier to DNA transformation in Caldicellulosiruptor species results in efficient marker replacement
Source: Biotechnol Biofuels. 2013 May 29;6:82. doi: 10.1186/1754-6834-6-82 (PMC3679861; doi:10.1186/1754-6834-6-82)
Supplement: Additional file 1: Figure S1 — Restriction endonuclease digests of chromosomal DNA isolated from Caldicellulosiruptor species. The nine restriction enzymes employed in this analysis are indicated on the top of the gel. (A) C. bescii chromosomal DNA. (B) C. saccharolyticus chromosomal DNA. M: 1 kb DNA ladder (NEB). Figure S2. Diagram of the cbeI (Cbes2438) knock-out vector. The gray colored boxes indicate sequences originating from C. bescii. Restriction sites and primers are indicated. aac, apramycin resistant gene cassette; pSC101, low copy replication origin in E. coli; repA and par, plasmid-encoded genes required for pSC101 replication and partition. Table S1. Primers used in this study. [file 1754-6834-6-82-S1.docx]

Supplemental Materials

Figure S1.


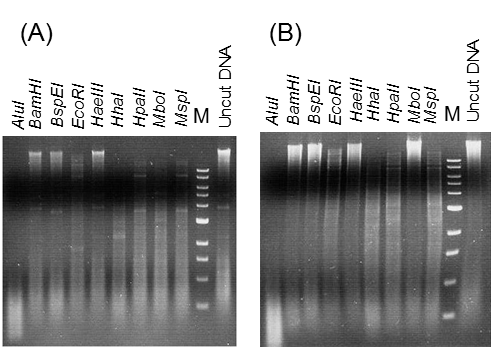


**Figure S1. Restriction endonuclease digests of chromosomal DNA isolated from *Caldicellulosiruptor* species**. The nine restriction enzymes employed in this analysis are indicated on the top of the gel. (A) *C. bescii* chromosomal DNA. (B) *C. saccharolyticus* chromosomal DNA. M: 1 kb DNA ladder (NEB).

Figure S2


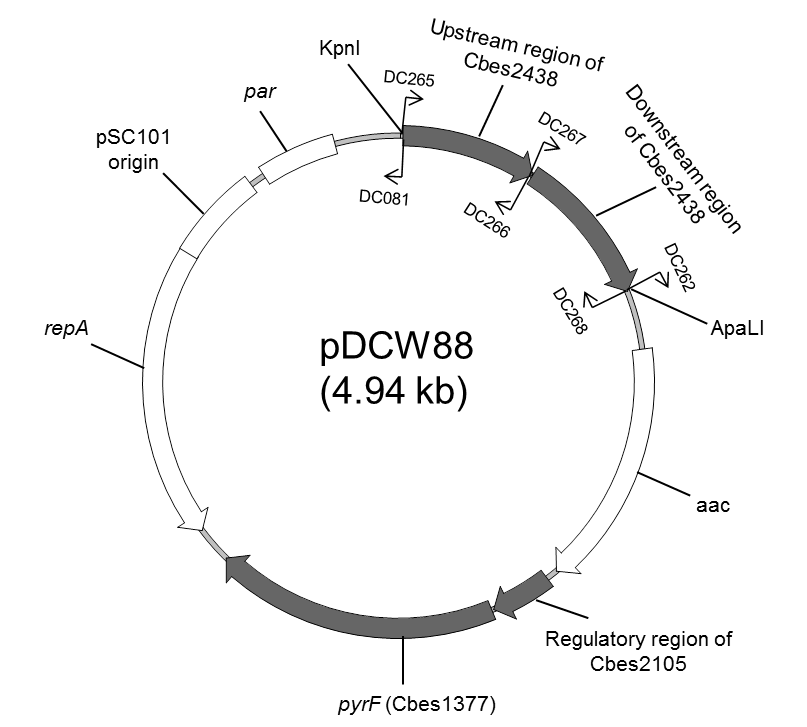


**Figure S2. Diagram of the *cbeI* (Cbes2438) knock-out vector.** The gray colored boxes indicate sequences originating from *C. bescii*. Restriction sites and primers are indicated. aac, apramycin resistant gene cassette; pSC101, low copy replication origin in *E. coli*; *repA* and *par*, plasmid-encoded genes required for pSC101 replication and partition.

Table S1. Primers used in this study.

| Primers | Sequences (5’ to 3’) | Description |
| --- | --- | --- |
| DC277 forward | TCTACACTCTTGCTTACACAGGT | To amplify the *cbeI* (Cbes_2438) region |
| DC239 reverse | TCTCCTCGAGCAGACCAAGTGCGTATTTTTC | To amplify the *cbeI* (Cbes_2438) region |
| DC265forward | AGAGAGGTACCTGCAACATCCGGCTTAATGAC | To amplify 440 bp of 5’ flanking region of (Cbes_2438) |
| DC266 reverse | TGTTAAAACCACCTACCTAATCTTATCATGTTGGAAGGCAAATTGA | To amplify 440 bp of 5’ flanking region (Cbes_2438) |
| DC267 forward | AGATTAGGTAGGTGGTTTTAACA | To amplify 487 bp of 3’ flanking region (Cbes_2438) |
| DC268 reverse | TGTGTGGTGCACTCCTTGATAATTTCAGCTGCCT | To amplify 487 bp of 3 ’flanking (Cbes_2438) |
| DC262 forward | TGTGTGGTGCACTCTGACGCTCAGTGGAACGAA | To amplify the *E.coli* features from pDCW 89 |
| DC081 reverse | AGAGAGGTACCACCAGCCTAACTTCGATCATTGGA | To amplify the *E.coli* features from pDCW 89 |
| JF263 forward | AGGTACCGGTTCATGTGCAGCTCCATC | To amplify the *aac* gene cassette |
| JF264 reverse | CTCCAACGTCATCTCGTTCTC | To amplify the *aac* gene cassette |
| DC100 forward | TAGTCTTGATGCTTCACTGATAG | To amplify the pSC101 *E. coli* replication origin |
| JF199 reverse | CGCTAACGGATTCACCACT | To amplify the pSC101 *E. coli* replication origin |
| DC233 forward | ATCCGTTGATCTTCCTGCAT | To amplify the *pyrF* cassette |
| DC235 reverse | AGGATCTGAGGTTCTTATGGCTC | To amplify the *pyrF* cassette |
